# Incidence and Clinical Outcomes of Vitamin D Status and Myeloma Bone Disease Among Patients With Newly Diagnosed Multiple Myeloma (MM) in a Tropical Country—Retrospective Cohort Study

**DOI:** 10.1002/cnr2.70431

**Published:** 2025-12-23

**Authors:** Chi Ching Lim, Wai San Wilson Tam, Widanalage Sanjay Prasad De Mel, Siew Ping Lang, Wee Joo Chng, Cinnie Yentia Soekojo, Fang Fang Song, Melissa Gaik Ming Ooi

**Affiliations:** ^1^ Division of Oncology Nursing National University Cancer Institute, Singapore Singapore; ^2^ National University Health System Singapore; ^3^ Department of Haematology‐Oncology National University Cancer Institute, Singapore Singapore

**Keywords:** incidence, multiple myeloma, skeletal morbidity, vitamin D

## Abstract

**Objective:**

The retrospective data analysis on 80 patients from 2018 to 2022 aimed to determine the incidence and clinical outcomes of vitamin D status and myeloma bone disease among newly diagnosed multiple myeloma (MM) patients in Singapore.

**Method:**

Patients' demographics, bone health data and bone disease management were collected. Chi‐square test was conducted to compare variables between patients who survived versus those who demised. Survival differences were compared using log‐rank analysis and plotted using Kaplan–Meier estimates. Multivariable survival analysis was conducted using Cox Regression.

**Results:**

Fifty‐nine (73.8%) patients had myeloma bone disease at diagnosis, and 42 (71.2%) of these patients had skeletal‐related adverse events at diagnosis. The compliance to bone health management needs to be optimized, as only 70 patients had their vitamin D level tested at diagnosis, of which 48 (68.7%) patients were vitamin D insufficient or deficient. Bone disease data did not correlate with mortality. Higher mortality was initially observed among patients with non‐Chinese (*p* = 0.03), R‐ISS 3 (*p* = 0.001), and renal involvement at diagnosis (*p* = 0.035). R‐ISS staging remained the only statistically significant variable after adjusting for race (adjusted HR 3.99; 95% CI 1.58–10.07).

**Conclusion:**

The study found no correlation between myeloma bone health data and survival outcomes in our centre.

## 1 Introduction

Multiple myeloma (MM) is the second most common type of blood cancer in Southeast Asian countries with more than 100 patients diagnosed in Singapore yearly [1]. It is a malignant plasma cell disorder characterized by clonal proliferation within the bone marrow and excessive production of monoclonal immunoglobulin. The disease course is heterogeneous, ranging from indolent to aggressive forms, and is frequently complicated by bone destruction, hypercalcemia, renal impairment, anaemia, and hypercalcemia. Notably, multiple osteolytic lesions are present in 80% of patients [2]. Importantly, survival outcomes have significantly improved over the last two decades with the emergence and transformation of novel therapeutic agents, including proteasome inhibitors, immunomodulatory drugs, and monoclonal antibodies [3]. These advances have transformed MM from a uniformly fatal condition to one with a median overall survival exceeding 5 to 10 years for many patients, particularly in countries where access to combination regimens and supportive care is optimized.

Vitamin D, a fat‐soluble steroidal hormone, plays a critical role in calcium homeostasis, bone mineralization, and immune modulation. In cancer biology, vitamin D is thought to influence cellular proliferation, differentiation, angiogenesis inhibition, and apoptosis, suggesting potential implications for disease progression and treatment outcomes [4]. Vitamin D deficiency has been widely reported in various malignancies, including MM, where it may contribute to bone fragility, pathological fractures, and skeletal‐related events (SREs) [5, 6, 7]. Furthermore, vitamin D insufficiency has been associated with reduced quality of life, fatigue, and increased symptom burden among cancer patients [8, 9, 10].

The relationship between vitamin D status and MM‐related bone disease remains controversial. Western studies have yielded mixed results: some demonstrated that low vitamin D levels are associated with more advanced disease at diagnosis, poorer prognosis, increased SREs and poorer transplant outcome [8, 11, 12, 13], while others report no significant correlation [5, 8, 11, 14, 15]. This variability may be attributed to differences in geographic location, genetic predisposition, treatment regimens, and methods of vitamin D assessment. However, data from tropical and Asian countries remain scarce. Despite Singapore's year‐round sunlight exposure, prior studies have documented a high prevalence of vitamin D deficiency among cancer patients, likely due to lifestyle factors such as limited outdoor activity, increased sunscreen use, and darker skin pigmentation reducing cutaneous synthesis [13].

Understanding vitamin D status in the context of MM is especially relevant in Southeast Asia, where cultural practices, diet, and environmental factors may differ significantly from Western populations. Moreover, bone disease is one of the most debilitating complications of MM, contributing to pain, reduced mobility, and diminished functional independence. Early identification and correction of vitamin D deficiency could represent a modifiable factor in optimizing bone health and potentially improving survival outcomes.

The objectives of this study were therefore twofold: (1) to determine the prevalence of vitamin D deficiency and myeloma bone disease in patients with newly diagnosed MM in a tropical country, and (2) to explore their associations with clinical characteristics and survival outcomes. By addressing this knowledge gap, we aim to inform regionally relevant strategies for bone health optimization and supportive care in MM management.

## 2 Materials and Methods

### 2.1 Study Design, Setting and Participants

This retrospective cohort study was conducted at a tertiary cancer centre that provides cancer treatments to predominantly the Western region of Singapore. This study is part of a major myeloma patient study in which patients who are 18 years and older with newly diagnosed disease between 2018 and 2022 were approached to participate in the study. Patients with monoclonal gammopathy of undetermined significance (MGUS), smoldering myeloma, and AL amyloidosis were excluded from study.

Patient demographics, disease characteristics, and treatment regimens were extracted from the hospital electronic medical record by the research coordinator independently and verified by the primary author of the study. Compliance to bone health management was defined as whether vitamin D level was assessed, supplementation initiated, and bone‐modifying agents commenced in accordance with institutional guidelines. During the study period, no standardized nursing‐led vitamin D replacement protocol was implemented, and treatment decisions were based on physician discretion. Some of the patients were referred to a myeloma advanced practice nurse for supportive care management post‐transplant, in which bone health is one of the core supportive care interventions after completion of the transplant. Revised‐International Staging System (R‐ISS) was used for myeloma staging [16]. Relevant imaging studies (Positron emission tomography (PET), Computerized tomography (CT), Magnetic resonance imaging (MRI), skeletal surveys, and X‐rays) were reviewed to determine the presence of bone disease when without complications, and skeletal‐related events (SREs) such as pathological fractures, spinal cord compression, and the need for radiation or surgery on bones at diagnosis.

### 2.2 Ethical Consideration and Informed Consent

This study is part of a major myeloma patient study and was approved by the National Healthcare Group Domain Specific Review Board (NHG‐DSRB, study reference number: 2012/00058‐AMD0014). Written consent was obtained from all participants via hardcopy consent form.

### 2.3 Serum 25‐Hydroxyvitamin D

Vitamin D status was assessed according to the Endocrine Society Clinical Practice Guideline (2011) and has the following range: vitamin D value of less than 20 ng/mL (50 nmol/L) is vitamin D deficiency, 21–29 ng/mL (52.5–72.5 nmol/L) is inadequate, and 30 ng/mL and over is adequate [17].

### 2.4 Myeloma Bone Disease and Skeletal Related Events

Myeloma bone disease is defined as the presence of lytic lesions due to myeloma when assessed using skeletal surveys, CT, PET, or MRI scans [18]. Skeletal‐related events are defined as the presence of vertebral compression fractures or long bone fractures at diagnosis using the above imaging studies [18].

### 2.5 Statistical Analysis

The primary hypothesis of the study was that newly diagnosed myeloma patients with low vitamin D level and delay in initiating bone modifying agents will have higher mortality. We referred to a similar study done in Australia for sample size calculation [15]. The study also examined correlation between vitamin D level and mortality rate, and 41 patients were recruited in the study. In our retrospective data analysis, we included 80 patients over 4 years. Mortality rate is compared between patients who were alive versus patients who have died by 31st July 2023. Categorical variables were compared using the chi‐square test, and results were presented as frequencies and percentages. Continuous variables were compared using Mann–Whitney *U* test and presented as median and interquartile range (IQR). Survival differences between patients who were alive versus dead were compared using log‐rank analysis and plotted as survival curves based on Kaplan–Meier estimates. Multivariable survival analysis was conducted using Cox regression to estimate survival probability using significant variables identified in log‐rank analysis earlier to adjust for covariation. Level of significance was set at 5%. All the computations were conducted using Jamovi version 2.3.20.

## 3 Results

### 3.1 Patient Characteristics

A total of 80 patients with newly diagnosed myeloma diagnosed in our centre between 2018 and 2022 were enrolled. Their demographic characteristics are summarized in Table 1. Out of 70 patients with their Vitamin D levels tested, 48 (68.7%) patients were vitamin D insufficient or deficient. Fifty‐nine (73.8%) patients had myeloma bone disease at diagnosis, and 42 (71.2%) of these patients with myeloma bone disease had skeletal‐related adverse events.

**TABLE 1:** Demographic, clinical characteristics, and status (death vs. alive).

Characteristics	Alive (*n* = 60)	Death (*n* = 20)	*p*
Age, median (IQR), years	65 (59–71)	69.5 (60–78.3)	0.201
Male sex, *n* (%)	33 (76.7)	10 (23.3)	0.698
Female sex, *n* (%)	27 (73)	10 (27)	
Race, *n* (%)			0.030*
Chinese	43 (82.7)	9 (17.3)	
Others (Malay, Indian, others)	17 (60.7)	11 (39.3)	
Clinical measurements			
R‐ISS staging, *n* (%)			0.001**
1	20 (90.9)	2 (9.1)	
2	29 (82.9)	6 (17.1)	
3	11 (47.8)	12 (52.2)	
Year of survival, median (IQR), years	2.71 (1.65–4.08)	1.54 (0.648–2.10)	< 0.001***
Bony involvement at diagnosis, *n* (%)			0.463
Yes	43 (72.9)	16 (27.1)	
No	17 (81)	4 (19)	
Skeletal related adverse events at diagnosis, *n* (%)			0.438
Yes	33 (78.6)	9 (21.4)	
No	27 (71.1)	11 (28.9)	
Renal involvement at diagnosis, *n* (%)			0.035*
Yes	20 (62.5)	12 (37.5)	
No	40 (83.3)	8 (16.7)	
Vitamin D status, *n* (%)			0.420
Sufficient	18 (81.8)	4 (18.2)	
Insufficient/deficient	35 (72.9)	13 (27.1)	
Not tested	7 (70)	3 (30)	
Started vitamin D replacement, *n* (%)			0.424
Yes	36 (72)	14 (28)	
No	24 (80)	6 (20)	
Started on bone modifying agents (e.g., zolendronic acid, denosumab, pamidronate), *n* (%)			0.649
Yes	45 (73.8)	16 (26.2)	
No	15 (78.9)	4 (21.1)	
Time to start BMAs, median (IQR), months	2 (1–4)	1.5 (1–4)	0.862
Types of bone modifying agents, *n* (%)			0.358
Zolendronic acid	36 (76.6)	11 (23.4)	
Others (Denosumab, Pamidronate)	9 (64.3)	5 (35.7)	
Did not start	15 (78.9)	4 (21.1)	
Started on calcium replacement, *n* (%)			0.260
Yes	16 (66.7)	8 (33.3)	
No	44 (78.6)	12 (21.4)	

*Note:* *p < 0.05; **p < 0.01; ***p < 0.001.

Abbreviations: IQR, interquartile range; R‐ISS, revised international staging system.

By 31st July 2023, 20 (out of 80) patients had died. When comparing patients who had demised against patients who survived, they were not statistically different in age, sex, and gender. However, patients with non‐Chinese race (*p* = 0.030), higher R‐ISS staging (*p* = 0.001), and patients with renal involvement at diagnosis of myeloma (*p* = 0.035) were more likely to have demised.

### 3.2 Survival Analysis

**FIGURE 1:**
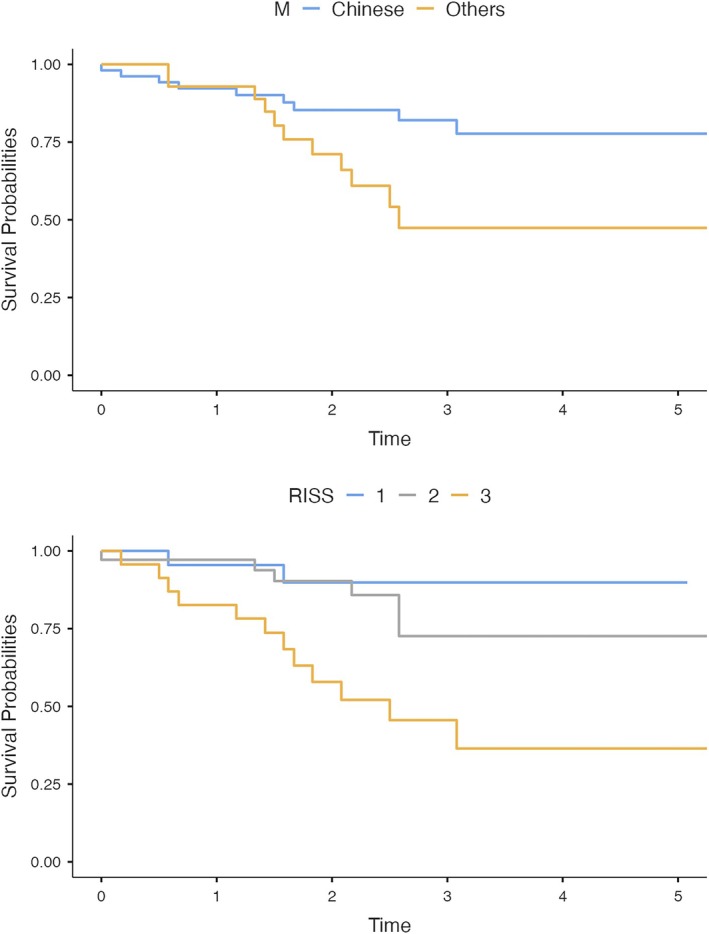
Survival Curves by race and Revised‐International Staging System (R‐ISS).

The results from survival analysis are summarized and presented in Table 2. The factors that are significant in survival analysis include race (*p* = 0.026) and R‐ISS staging (*p* < 0.001), with survival curves illustrated as Kaplan–Meier estimates in Figure 1 [19, 20].

**TABLE 2:** Survival analysis using log‐rank test.

Characteristics	Median survival time (IQR)	*p*
Gender		0.939
Male	2.17 (1.25–2.88)	
Female	2.58 (1.75–4.25)	
Race		0.026*
Chinese	2.54 (1.46–3.79)	
Others (Malay, Indian, others)	1.96 (1.90–4.83)	
Clinical measurements		
R‐ISS staging		< 0.001***
1	3.33 (1.77–4.44)	
2	2.17 (1.50–3.00)	
3	1.83 (1.29–2.67)	
Presence of bone disease		0.295
Yes	2.17 (1.25–3.17)	
No	2.75 (2.08–4.25)	
Baseline skeletal related event at diagnosis		0.550
Yes	2.17 (1.42–3.46)	
No	2.29 (1.46–3.88)	
Renal disease at diagnosis		0.056
Yes	2.17 (1.48–3.12)	
No	2.46 (1.29–4.02)	
Started bone modifying agents		0.767
Yes	2.25 (1.50–3.67)	
No	2.17 (1.17–3.67)	
Vitamin D level (sufficient vs. insufficient/deficient)		0.471
Yes	2.29 (1.44–3.42)	
No	2.17 (1.42–3.69)	
Started vitamin D replacement		0.519
Yes	2.17 (1.50–3.50)	
No	2.25 (1.35–3.98)	
Started calcium replacement		0.442
Yes	2.54 (1.65–3.62)	
No	2.17 (1.33–3.67)	

*Note:* *p < 0.05; ***p < 0.001.

Abbreviations: IQR, interquartile range; R‐ISS, revised international staging System.

Multivariable survival analysis was then conducted using statistically significant factors from the earlier survival analysis, as illustrated in Table 3 [21]. It is revealed that only R‐ISS staging remained as the only statistically significant variable for survival.

**TABLE 3:** Multivariable survival analysis using cox regression.

Characteristics	All (*n* = 80)	HR (multivariate)	*p*
R‐ISS staging			
R‐ISS 1 + *R*‐ISS 2	57		
R‐ISS 3	23	3.99 (1.58–10.07)	0.003**
Race			
Chinese	52		
Others (Malay, Indian, others)	28	1.94 (0.78–4.83)	0.156

*Note:* **p < 0.01.

Abbreviations: IQR, interquartile range; R‐ISS, revised international staging system.

## 4 Discussion

Singapore is a multiracial and multi‐cultural country consisting of a vastly diverse genetic make‐up with its predecessors coming from various parts of Asian countries. The main race of Singapore is Chinese. To our knowledge, this study is the first attempt to examine the incidence of bone disease and bone health status among newly diagnosed Singaporean myeloma patients, and its correlation with their clinical outcomes. Notably, over 70% of patients with newly diagnosed myeloma in our centre have bone involvement at diagnosis, of which over 70% of these patients suffer from skeletal‐related adverse events at diagnosis.

In this study, we observed a high prevalence (68.5%) of vitamin D insufficiency, deficiency and bone disease among patients with newly diagnosed MM despite the tropical environment. Our finding is similar to a recent study completed in a similar tropical region of Townsville in Australia and in Mayo Clinic in the States [7, 15]. Vitamin D deficiency seems to be prevalent among myeloma patients regardless of the amount of potential sun exposure [7]. To determine if bone health data correlates to patients' clinical outcomes, we examined if the vitamin D status, use of bone modifying agents and calcium and vitamin D replacement could augment their mortality rate. Contrary to earlier studies, we do not find an association between the above bone health data and patients' mortality [13, 22, 23]. This could be contributed to by small sample size, retrospective design, limited longitudinal monitoring, and potential timing delays in intervention initiation among patients which may have attenuated measurable effects on survival.

When comparing the demographic data between patients who survived versus those who have demised, patients' race (non‐Chinese), renal involvement at diagnosis, and R‐ISS staging are statistically significant. This is despite Chinese being the main racial group in a multi‐racial Singapore society and constituting over 75% of the Singapore population [24]. While our study found a higher mortality rate among non‐Chinese patients initially in univariate analysis, it was not an independent predictor after adjustment for confounders in multivariate analysis, and this observation must be interpreted with caution. Potential confounding factors, such as socio‐economic status, healthcare accessibility, cultural differences in health‐seeking behaviors, and underlying genetic variations, may have contributed to this finding. As our study did not adjust for these variables, the association between race and survival outcomes should be viewed as exploratory. Larger, prospective studies accounting for these factors are needed to clarify the relationship between race and prognosis in multiple myeloma. Renal impairment is a common disease complication that myeloma patients face, primarily due to excessive production of monoclonal chains [25]. Our study result is congruent with international studies, as impaired renal function has been associated with poorer survival outcomes when compared to patients without renal impairment at diagnosis, even if renal function improves after treatment [26]. Our patients with higher R‐ISS staging, especially stage III, tend to have poorer survival, similar to international studies conducted.

When survival analysis using log‐rank test was conducted, only R‐ISS and race remained statistically significant. Finally, when multivariable survival analysis was conducted using Cox regression, only R‐ISS staging remained significant. As such, R‐ISS staging remains the most significant factor in determining the survival outcomes among our patients.

We note that the compliance to bone health screening and management among patients can be further optimized, as vitamin D level was not screened in 10 (12.5%) patients, and 19 patients (23.8%) were not started on bone‐modifying agents. This result indicates the need to deep dive into the barriers to bone health screening and non‐compliance to bone‐modifying agents, and the need to develop a structured survivorship program in which specialty oncology nurses' role could potentially address the gaps, especially at the stage when patients are newly diagnosed with MM.

## 5 Study Limitations

Our study has a few limitations. Firstly, although myeloma is the second most common hematologic malignancy in Singapore, it is still rare and the number of newly diagnosed patients remains small at about 100 annually. As such, the sample size of our study is relatively small. Secondly, bone health data such as vitamin D level is not mandated as part of routine investigations for newly diagnosed myeloma patients, resulting in a few patients not screened or managed for vitamin D deficiency. Variability in clinicians' bone health practice patterns may have influenced outcomes. Thirdly, there is a lack of information on longitudinal vitamin D monitoring and effects of vitamin D supplementation. Lastly, the study is a retrospective cohort study; thus, the study could correlate factors with mortality, but there is no causality interpretation. As such, the findings should be interpreted within the context of this limitation.

## 6 Conclusion

In this study, we found that despite Singapore's tropical climate, vitamin D insufficiency remained highly prevalent among newly diagnosed Southeast Asian myeloma patients. We found no correlation between myeloma bone health data and survival outcomes, which could be attributed to variations in patient demographics, lifestyle factors such as sun exposure habits, dietary intake, genetic backgrounds, or differences in healthcare practices between regions. This comparison underscores the importance of considering regional and cultural factors when evaluating the role of vitamin D in disease progression and outcomes in multiple myeloma. Prospective studies have to be done to include more patients from the minority racial group in Singapore to examine if race correlates with disease outcomes such as survival rates and disease progression. Further studies are also needed to explore if optimizing myeloma bone disease management could reduce the risk of further skeletal‐related events and its impact on quality of life among our patients.

### 6.1 Relevance for Clinical Practice

The high incidence of myeloma bone disease and vitamin D deficiency among our patients in a tropical country of Singapore warrants the need to implement guidelines on the use of bone‐modifying agents and include vitamin D screening and in newly diagnosed myeloma patients. Myeloma specialty nurse‐led bone health care plans should be explored as a feasible intervention to optimize compliance and bone health outcomes for patients, initiating at the point of diagnosis and throughout the patient treatment trajectory.

## Author Contributions

Chi Ching Lim, Wai San Wilson Tam, Widanalage Sanjay Prasad De Mel, Siew Ping Lang, Wee Joo Chng, Cinnie Yentia Soekojo, Fang Fang Song, and Melissa Gaik Ming Ooi conceptualized and designed the study; Chi Ching Lim and Wai San Wilson Tam analyzed the data; all authors contributed to the interpretation of the data. Chi Ching Lim drafted the article; all authors revised the article critically for important intellectual content. All authors approved the final version of the article to be published. All authors agree to be accountable for all aspects of the work in ensuring that questions related to the accuracy or integrity of any part of the work are appropriately investigated and resolved.

## Funding

The study received funding from Yong Siew Yoon Research Fellowship, which covers the publishing fee for this article.

## Disclosure

Associate Professor Wilson Tam is the statistician on the author team. The authors affirm that the methods used in the data analyses are suitably applied to their data within their study design and context, and the statistical findings have been implemented and interpreted correctly. The authors agree to take responsibility for ensuring that the choice of statistical approach is appropriate and is conducted and interpreted correctly as a condition to submit to the Journal.

## Conflicts of Interest

The authors declare no conflicts of interest.

## Data Availability

Participants of the research study were assured that raw data would remain confidential and would not be shared. As such, the data would not be available and is confidential.
